# Wear and Friction Analysis of Brake Pad Material Using Natural Hemp Fibers

**DOI:** 10.3390/polym15010188

**Published:** 2022-12-30

**Authors:** Mithul Naidu, Ajit Bhosale, Yashwant Munde, Sachin Salunkhe, Hussein Mohamed Abdelmoneam Hussein

**Affiliations:** 1 Department of Mechanical Engineering, Sinhgad College of Engineering, Savitribai Phule Pune University, Pune 411041, India; 2 Department of Mechanical Engineering, MKSSS’s Cummins College of Engineering for Women, Pune 411052, India; 3 Department of Mechanical Engineering, Vel Tech Rangarajan Dr. Sagunthala R&D Institute of Science and Technology, Chennai 600062, India; 4 Mechanical Engineering Department, Faculty of Engineering and Technology, Future University in Egypt, New Cairo 11835, Egypt; 5 Mechanical Engineering Department, Faculty of Engineering, Helwan University, Cairo 11732, Egypt

**Keywords:** hemp fibers, polymer composite, friction, wear, Taguchi optimization, analysis of variance

## Abstract

Non-exhaust brake dust and pollution arising from metal, semi-metal, and ceramic brake pads have made recent research consider their replacement by potential natural fibers such as hemp, flax, sisal, etc. These natural fibers are lightweight, biodegradable, and cheap. This paper discusses the wear and friction analysis of hemp fiber reinforced polymer brake pad material. Three test specimens viz. HF4P20, HF5P20, and HF6P20 were prepared per ASTM G99 standards for the pin-on disc tribo-test. The test trials and validation were done using the Taguchi design of experiments and ANOVA. The optimum result showed a consistent coefficient of friction and lowered specific wear rate for HF6P20 brake pad material. Worn surface morphology was done using scanning electron microscopy.

## 1. Introduction

Alternatives of non-asbestos organic friction materials for brake pad applications, after the global ban on asbestos fibers in the late ’80s, because of its carcinogenic effects on the environment, had promoted the use of synthetic fibers for the same [1,2].

However, with the increase in the consumption of synthetic/artificial fiber reinforced polymers (FRP), environmental concerns relating to the limited recyclability and end-of-life disposal options of FRPs have been highlighted. The perceived scale of the problem had even led to strict government legislation, such as the EU directive on the landfill of waste (Directive 1999/31/EC) and the End-of-life Vehicle Directive (Directive 2000/53/EC), which were seen as barriers to the development and continue the use of FRPs in market. In addition, the high cost of synthetic fiber such as glass, carbon, and aramid resulted in a high cost of production and products derived from these materials, which necessitated alternative means of friction material development.

Natural fibers thus emerged as a renewable and cheaper substitute for synthetic materials such as glass fibers, carbon fibers, and aramid, which are used as reinforcement in brake pad materials [3,4]. The use of natural fiber composites (NFC) is growing for many reasons, including their potential to replace synthetic fiber-reinforced plastics at a lower cost with improved sustainability [5].

There are different types of natural fibers, such as bast fibers, leaf fibers, grass/reed fibers, and seed fibers. These bast fibers have better properties such as tensile stiffness and specific tensile strength than others. Bast fiber includes flax, jute, hemp, kenaf, and ramie [6].

Industrial hemp fiber is one of the strongest and stiffest available bast fiber and therefore has a great potential as a reinforcement in composite material. The use of hemp fiber in the composite industry as reinforcement is its high specific strength, low weight, low cost of production, and eco-friendliness.

This paper discusses the development and tribological analysis of three variants of hemp fiber reinforced polymer brake pad compositions. HF4P20, HF5P20, and HF6P20, using the Taguchi design optimization technique. Analysis of variance (ANOVA) was also performed to understand the significant contributions of the factors influencing friction and wear performances. Worn surface micrographs were studied by scanning electron microscopy (SEM). However, the authors aim to use the hybrid CRITIC-MEW approach as an optimization tool for selecting the best-performing brake friction composite in their future work [7].

The results reveal HF6P20 to exhibit itself as a better brake friction material with a lower specific wear rate (SWR) and a moderate but consistent coefficient of friction (COF) within the levels prescribed by earlier studies [8,9,10] and by SAE J2986 standards for passenger car brakes. 

## 2. Materials and Methods

### 2.1. Fabrication of Compositions

Hemp fibers procured from Hemp Affair Pvt. Ltd. in Varanasi (Noida, India) were chemically processed for 24 h in 4%, 5%, and 6% NaOH solution (*w*/*v*) [11,12,13], washed with distilled water and then dried for 10 h in sunlight. After that, the hemp fibers were chopped into 3–5 mm lengths. The ingredients contained, Barium sulfate as a filler [8,11], fibers as reinforcement, phenol-formaldehyde as a binder [11,14], graphite as a dry lubricant [8,14], vermiculite and alumina as properties modifiers [8,11], HF4P20—with 4% NaOH treated hemp fibers, HF5P20—with 5% NaOH treated hemp fibers, and HF6P20—with 6% NaOH treated hemp fibers were made employing these ingredients. Table 1 shows the weight percentages of the elements in the mixtures.

All the ingredients were measured digitally (Wensar^®^ weighing scales Ltd., Chennai, India, Range 0–220 g, least count 0.01 g). The chopped fibers and phenolic powder formulation were mixed for 15 min at 250 to 500 rpm in a mechanical stirrer to achieve a homogenous mixture. The mixtures were compressed in a compression molding machine (Santec Inc., Delhi, India). The mixes were cured for 10 min each with four breathings at 15 MPa and 155 degrees Celsius. For 3 h, the post-curing was done in a hot air oven (Athena technology, Mumbai, India) at 170 °C. [8], to remove moisture and trapped gases formed during the matrix constituents’ polymerization process and release the induced compressive stresses.

**Figure 1:**
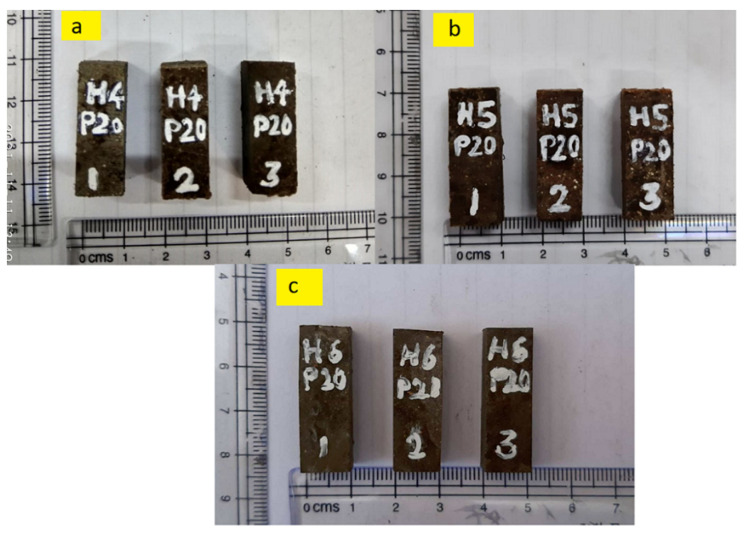
(**a**) HF4P20, (**b**) HF5P20, (**c**) HF6P20.

Plates of three compositions, namely, HF4P20, HF5P20, and HF6P20 of dimensions 100 × 100 × 10 mm thick, were prepared using the compression molding technique. Specimens for Pin-on disc test as per ASTM G 99 were derived from 100 × 100 × 10 mm plates of each type of composition, as shown in Figure 1a–c.

### 2.2. Friction and Wear Testing

#### 2.2.1. Design of Experiments using Orthogonal Array

Friction properties such as specific wear rate (SWR) and coefficient of friction (COF) were analyzed using Taguchi’s experimental design [15]. Test trials and combinations of these properties were selected according to Taguchi standard L9 orthogonal array for HF4P20, HF5P20, and HF6P20 [16]. Configuration, load, and sliding speed were the considered factors influencing SWR and COF. As shown in Table 2, three levels were selected for each factor to understand the results clearly. These test parameters were chosen from previous studies. [1,10,17].

#### 2.2.2. Experimental Procedure

To evaluate the geometrical performance of the five compositions, they were tested on a Pin-on Disc tester (DUCOM™ TR20LE, Bohemia, NY, USA) according to ASTM G 99. 100 mm track diameter for 5000 m sliding distance was selected. Various combinations were obtained using the Taguchi L9 design for three factors, and three levels were tested. The obtained COF and SWR results are presented in Table 3.

## 3. Results and Discussion

Taguchi Design of Experiments (DOE) was conducted with three factors—composition, load, and sliding velocity, and each factor with three varying levels. L9 orthogonal array was suggested by Taguchi design of experiment method for three factors and three levels. The derived experiments were used for tribological testing on a Pin-on disc tribo-machine as per ASTM G99. Subsequently, Specific Wear Rate (SWR) and Coefficient of Friction (COF) were obtained using Equations (1) and (2) as shown below and analyzed using S/N ratios to decide the optimum parameters.

(1) $$SWR=\frac{∆m}{\rho LD}$$

(2) $$COF=\frac{F}{L}$$

where, ∆m = weight loss, ρ = density, *L* = applied load, *D* = sliding distance, *F* = frictional force.

### 3.1. Signal-to-Noise Ratio 

Specific Wear Rate (SWR) and Coefficient of Friction (COF) are converted into S/N ratios. SWR and COF should be ideally minimum. Hence the formula for the ‘Smaller is Better’ quality characteristic is applied. The formula to calculate the respective S/N ratios for SWR and COF is Equation (3), as shown below.

(3) $$\frac{\;S}{N}ratio=-10\log_{10}\{\frac{1}{n}\overset{n}{\underset{i=1}{\sum }}y_{i}^{2}\;\;\}$$

where, *y* = SWR or COF and *n* = number of trials.

The purpose is to compute the highest signal-to-noise ratio, which means minimum random factors (noise) affect the required parameters. The values of S/N ratios are tabulated in Table 3.

### 3.2. Variation of Friction Force (F), SWR and COF with Respect to Normal Load

**Figure 2:**
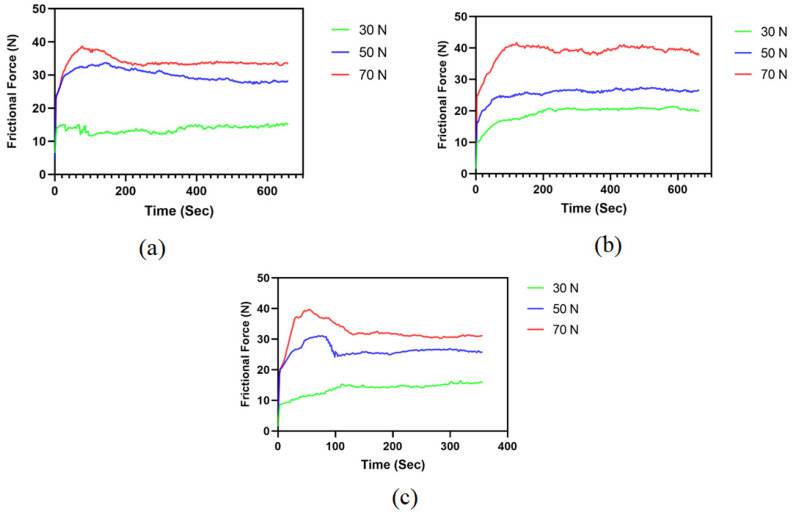
Variation of friction force with respect to time for (**a**) HF4P20, (**b**) HF5P20, and (**c**) HF6P20.

Variation of friction force concerning time was recorded for all three compositions at 30 N, 50 N and 70 N, as shown in Figure 2a–c.

The abrasive wear of the brake pad is principally manifested as a “plowing effect”. The massive particles or small bulges penetrate and scrape the brake pad material; wear scars and abrasive dirt are thus made on the surface. The hard particles are pressed into the contact surface of the friction pair under load generating indentation that will increase the surface roughness of the friction pair, in order that the contact peak of the micro bulge is additional doubtless to create a bond point, and therefore the adhesive wear of the brake pad occurs. Especially under high speed and heavy-load conditions, due to the large plastic deformation of the contact peak and high surface temperature, the phenomena of the shear in the bond point of fracture are caused by the relative sliding of the brake friction pair surface. Shedding materials become abrasive dust; others migrate from the brake surface to the disc surface to wear continually [18]. This phenomenon has been revealed and validated in Figure 2a–c showing typically two friction regimes, initially a running-in period, followed by a steady-state period [1,19].

**Figure 3:**
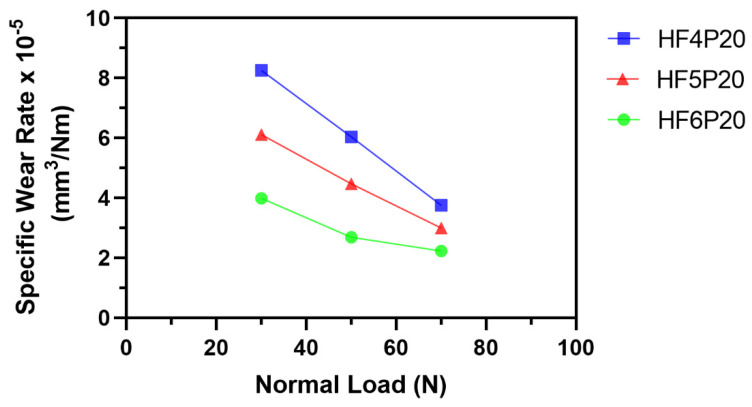
Variation of specific wear rate with respect to normal load.

**Figure 4:**
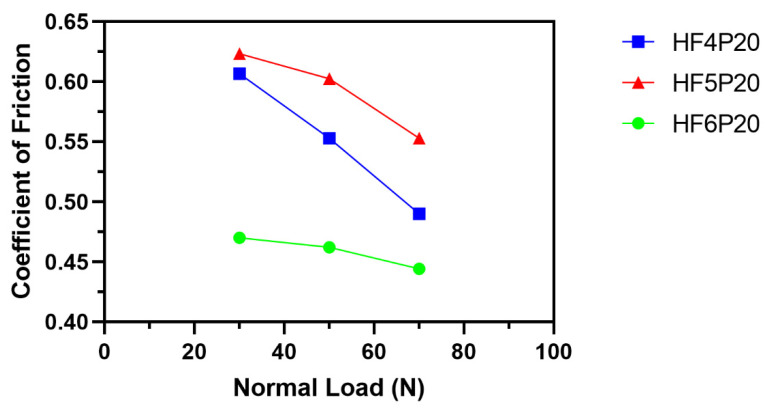
Variation of coefficient of friction with respect to normal load.

The initial regime of the running-in period was due to the higher initial adhesive forces between the test materials and the metal rotor due to the adhesive wear phenomenon as discussed, followed by a steady-state period showing consistent friction forces concerning time for all three normal load conditions observed in HF4P20, H5P20 and HF6P20, as shown. The second regime reveals the stability in the abrasive wear, which confirms the consistency of the friction forces in this regime. Friction force trends show a direct relation to the normal loads acting on the friction surface. Friction forces increase with the increase in normal loads. The SWR and COF values were plotted at three different average load values, 30 N, 50 N, and 70 N, as shown in Figure 3 and Figure 4. The sliding speed was not considered in this case because of its negligible influence on the response behavior, as shown in Table 4 and Table 5.

Figure 3 shows Specific Wear Rate (SWR) of HF6P20 is on the lower side due to good interfacial bonding between fibers and matrix than in the cases of HF4P20 and HF5P20. This strong interfacial bonding might be due to alkali pretreatment, which etches the fiber surface, removing the wax and impurities on them [19]. The drop in the SWR with the rise in the normal load from 30 N to 70 N is expected, as seen for all three compositions here. This is because, as per Equation (1), SWR is inversely proportional to the normal load, and also, the wear type at the initial stage is likely to be adhesive type, which gradually changes to abrasive type with an increase in the asperity contact temperatures at the interface of the friction composites and the metal counter-face [20].

In Figure 4, COF values for all three compositions show a decreasing trend with the increase in the normal load from 30 N to 70 N, following the mathematical relation shown in Equation (2). This might be due to worn surface modification due to the transfer layer that might have formed on the friction surface. In addition, the rise in asperity contact temperature at higher loads could be another possible reason for the drop in COF [19]. Amongst these compositions, HF6P20 shows lower and stable values of COF due to better fiber-matrix interfacial bonding compared with its counterparts, i.e., HF5P20 and HF4P20.

### 3.3. Analysis of Variance

Analysis of variance (ANOVA) is a statistical process to acquire the contribution of composition, load, and velocity in the performance characteristics: SWR and COF. The significance of each control factor has been determined using ANOVA by comparing the F values of each control factor. The greater the F contribution, the greater the impact of a factor on the result. The percentage contribution of each factor to total variation is shown in the last column of the ANOVA tables, indicating the degree of impact on the results. The assigned factor is statistically and physically insignificant when ‘F’ is less than the ‘5%’ [21]. Table 4 and Table 5 shows the ANOVA results for SWR and COF, respectively. The percentage contribution of factors is also the ratio of the Sum of Squares of the factor to the Total Sum of Squares which gives similar results as those revealed by the F values. For SWR, normal load plays the most significant role (54.50%), followed by composition (42.12%), and finally, by sliding velocity of the disc (2.02%) whereas for COF, composition has highest contribution (74.00%), followed by the normal load (24.19%), and lastly the sliding velocity (0.04%).

### 3.4. Optimization of Factors

**Figure 5:**
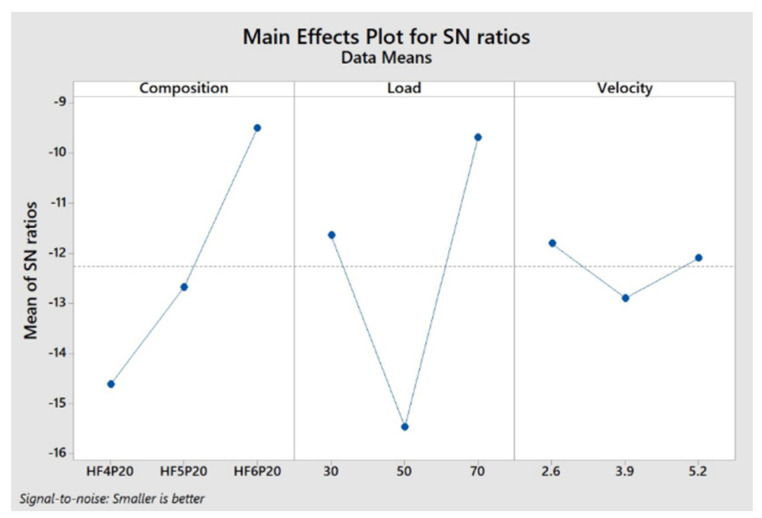
Main effects plot for SWR.

**Figure 6:**
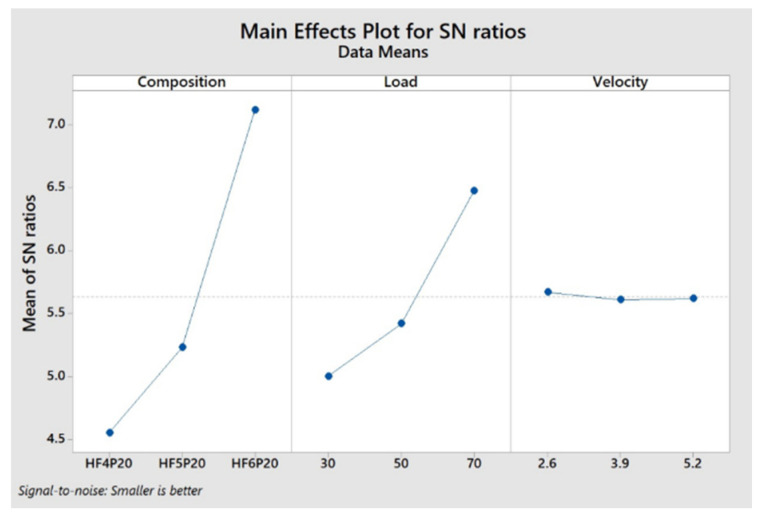
Main effects plot for COF.

The optimum levels suggested by the Taguchi optimum design for the factors of Specific Wear Rate and Coefficient of Friction were obtained from respective main effects plots for S/N ratios. Figure 5 and Figure 6 represent optimum factor levels for SWR and COF. Both Figure 5 and Figure 6 show the composition of HF6P20, applied load of 70 Newton, and sliding velocity of 2.6 m/s as an optimum combination for optimum SWR and COF, respectively. Table 6 and Table 7 show rank-wise contribution of factors influencing SWR and COF, respectively, by Taguchi design. These contribution levels of factors agree with those indicated by ANOVA, as shown in Table 4 and Table 5, respectively. The optimum factors have been tabulated in Table 8.

### 3.5. Experimental Validation of Optimum Factors

Optimum SWR and COF values were predicted by the Taguchi method as shown in Figure 5 and Figure 6, and regression Equations (4) and (5) were experimentally verified and tabulated as shown in Table 9. The predicted and actual values of SWR and COF are within the acceptable limit of 10% error, hence valid. However, the error in the predicted and the actual values of SWR and COF might be due to the increase in the amplitude of mechanical vibrations at elevated speeds in the experimental setup. This is because the average contact area of the two sliding objects is reduced with respect to the increase in the amplitude of vibration, as the distance between the vibrating surfaces in contact is increased [22]. Table 9 reveals the same, showing a drop in the actual values of SWR and COF compared with their predicted values using Equations (4) and (5), respectively.

SWR = 5.00 − 0.0213 Load + 0.125 Velocity(4)

COF = 0.638 − 0.00219 Load + 0.00 Velocity(5)

### 3.6. Worn Surface Morphology

Scanning Electron Microscope (Zeiss EVO^®^ MA 15, Zeiss, Oberkochen, Germany) was used to obtain the micrographs of the worn surfaces of the test specimens tested on Pin-on disc setup for HF4P20, HF5P20, and HF6P20 at 70 N load. The wear mechanism of polymer matrix composites can be described by different modes such as fiber pullouts, fiber-matrix de-bonding, matrix debris formation, matrix crack, and contact plateau formation.

**Figure 7:**
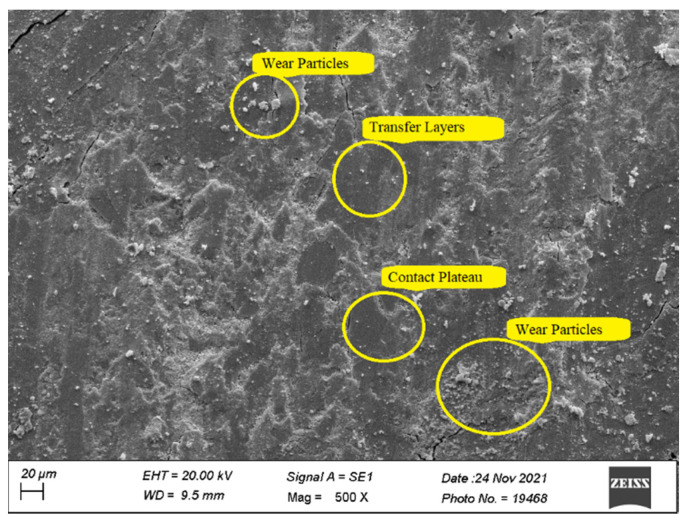
Worn surface micrograph of HF4P20.

Figure 7 shows the micrograph of HF4P20, where a large number of wear particles was observed. This might be due to poor interfacial fiber-matrix bonding due to a lower concentration of alkali treatment given to the hemp fiber of 4% (*w*/*v*) compared with its counterparts. In addition, a considerable amount of secondary contact plateaus was observed due to the compaction of the wear debris, giving rise to a rise in SWR (Figure 3) as compared with its counterparts studied here [23]. Non-coherent transfer layers, which are quite evident, might have resulted in the COF of HF4P20 being lower than HF5P20 but higher than HF6P20, as evident in Figure 4 [1].

**Figure 8:**
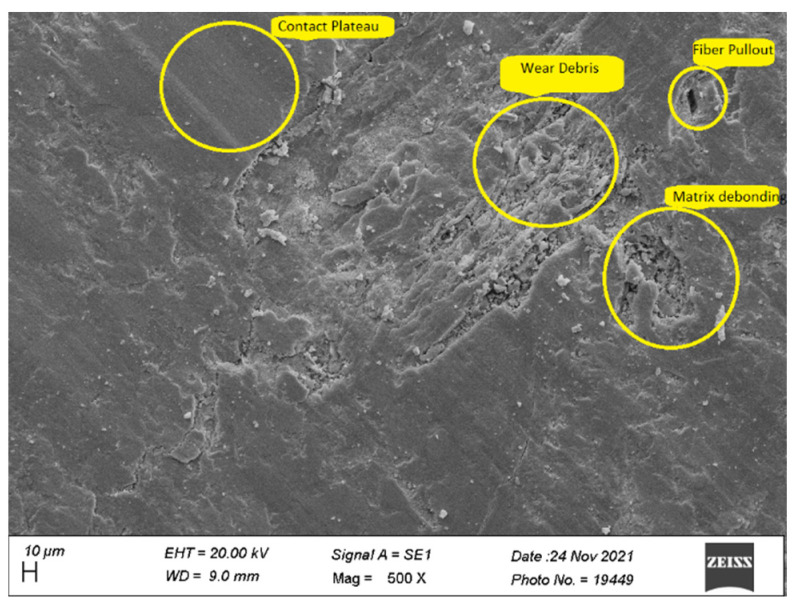
Worn surface micrograph of HF5P20.

Figure 8 shows the micrograph of HF5P20 where matrix de-bonding and fiber pullout were observed. This was due to poor interfacial fiber-matrix bonding compared with HF6P20, due to a lower concentration of alkali treatment given to the hemp fiber of 5% (*w*/*v*) compared with HF6P20. In addition, a considerable amount of wear debris was observed due to loose fiber-matrix bonding resulting from the observed SWR (Figure 3). Coherent transfer layers, compared with HF4P20, are quite evident; that might have resulted in the m COF for HF5P20 being higher than HF4P20 and HF6P20, as shown in Figure 4.

**Figure 9:**
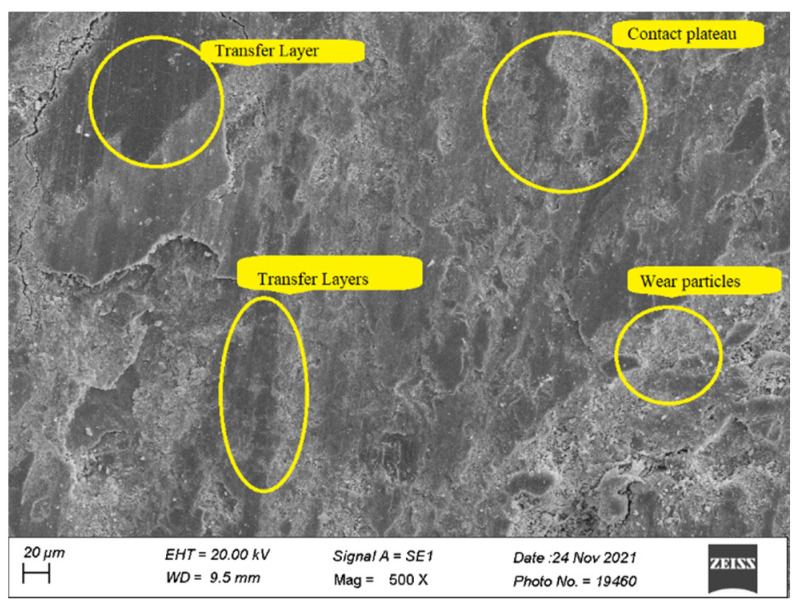
Worn surface micrograph of HF6P20.

Figure 9 shows the micrograph of HF6P20 where consistent and large contact plateaus were seen, which might have reduced the SWR and maintained a lower but consistent and acceptable value of COF. This was due to improved interfacial fiber-matrix bonding compared with HF4P20 and HF5P20, which resulted from a higher concentration of alkali pre-treatment given to the hemp fiber of 6% (*w*/*v*). A lesser amount of wear debris was observed due to improved fiber-matrix bonding, resulting in the observed SWR (Figure 3). Thin significant transfer layers kept the COF lower and desirably consistent [24].

## 4. Conclusions

Three variants of bio-composite brake pad materials, HF4P20, HF5P20, and HF6P20, were prepared and tested for friction and wear parameters using the Taguchi design of experiments following the ASTM G99 standard, wherein the following conclusions were made.

1. HF6P20 was a better bio-friction material by exhibiting lower SWR and lower but consistent and acceptable COF values than its counterparts studied here.
2. Taguchi design and ANOVA results suggested ‘composition’ as the most significant factor for COF and ‘normal load’ for SWR for all three compositions.
3. Experimental results of optimum SWR and COF values showed close agreement with those predicted by the regression model.
4. SEM also revealed consistent contact plateaus and coherent transfer layers for HF6P20 than its counterparts studied here.

## Figures and Tables

**Table 1:** Material compositions.

Materials	Weight Contribution (%)		
	HF4P20	HF5P20	HF6P20
Hemp fibers	25	25	25
Phenol Formaldehyde	20	20	20
Graphite powder	5	5	5
Vermiculite	5	5	5
Alumina	5	5	5
Barium sulphate	40	40	40
**Total**	**100**	**100**	**100**

**Table 2:** Factors and levels.

Factors	Units	Level 1	Level 2	Level 3
Composition	-	HF4P20	HF5P20	HF6P20
Load	N	30	50	70
Velocity	m/s	2.6	3.9	5.2

**Table 3:** Specific wear rate (SWR) and coefficient of friction (COF) using L9 orthogonal array.

Composition	Load (N)	Velocity (m/s)	SWR × 10^−5^ (mm^3^/Nm)	COF	S/N Ratio (SWR)	S/N Ratio (COF)
HF4P20	30	2.6	6.111	0.6066	−18.03619344	4.341951881
HF4P20	50	3.9	4.467	0.5526	−14.33697165	5.148639313
HF4P20	70	5.2	2.9917	0.4900	−11.48062535	6.196078399
HF5P20	30	3.9	8.2528	0.6233	−16.27894954	4.106057462
HF5P20	50	5.2	6.0375	0.6024	−12.21405722	4.402300736
HF5P20	70	2.6	3.75	0.5528	−9.518360829	5.148639313
HF6P20	30	5.2	3.9893	0.47	−11.59278503	6.558042841
HF6P20	50	2.6	2.6909	0.462	−8.84131341	6.707160489
HF6P20	70	3.9	2.2298	0.444	−8.061723765	8.083464459

**Table 4:** ANOVA for S/N ratio of specific wear rate.

Source	DF	Seq SS	Adj SS	Adj MS	F	P	Contribution (%)
Composition	2	40.061	40.061	20.0304	31.24	0.031	42.12
Load	2	51.830	51.830	25.9148	40.42	0.024	54.50
Velocity	2	1.920	1.920	0.9599	1.50	0.400	2.02
Error	2	1.282	1.282	0.6412	-	-	
Total	8	95.093	-	-	-	-	

Seq. SS = Sequential Sum of squares, Adj. SS = Adjusted Sum of Squares, Adj. MS = Adjusted Mean Square, DF = Degree of freedom F = Ratio of explained variance to unexplained variance, P = Probability of obtaining F value.

**Table 5:** ANOVA for S/N ratio of coefficient of friction.

Source	DF	Seq SS	Adj SS	Adj MS	F	P	Contribution (%)
Composition	2	10.5933	10.5933	5.29664	42.32	0.023	74.00
Load (N)	2	3.4636	3.4636	1.73179	13.84	0.067	24.19
Velocity (m/s)	2	0.0064	0.0064	0.00319	0.03	0.975	0.04
Error	2	0.2503	0.2503	0.12515	-	-	
Total	8	14.3135	-	-	-	-	

Seq. SS = Sequential Sum of squares, Adj. SS = Adjusted Sum of Squares, Adj. MS = Adjusted Mean Square, DF = Degree of freedom F = Ratio of explained variance to unexplained variance, P = Probability of obtaining F value.

**Table 6:** Response Table for Signal-to-Noise Ratios for SWR.

Level	Composition	Load	Velocity
1	−14.618	−11.636	−11.799
2	−12.670	−15.464	−12.893
3	−9.499	−9.687	−12.096
Delta	5.119	5.777	1.094
Rank	2	1	3

(Smaller is better).

**Table 7:** Response Table for Signal-to-Noise Ratios for COF.

Level	Composition	Load	Velocity
1	4.552	5.002	5.670
2	5.229	5.419	5.609
3	7.116	6.476	5.618
Delta	2.564	1.474	0.061
Rank	1	2	3

(Smaller is better).

**Table 8:** Optimum factors as per main effect plots for S/N ratio.

Parameters	Composition	Load (N)	Velocity (m/s)
SWR	HF6P20	70	2.6
COF	HF6P20	70	2.6

**Table 9:** Experimental validation of optimum factors.

Particulars	Specific Wear Rate		Particulars	Coefficient of Friction	
	Actual	Predicted		Actual	Predicted
**Factor Level**	L: 70 N	L: 70 N	**Factor Level**	L: 70 N	L: 70 N
	V: 2.6 m/s	V: 2.6 m/s		V: 2.6 m/s	V: 2.6 m/s
	C: HF6P20	C: HF6P20		C: HF6P20	C: HF6P20
**SWR**	3.5417 × 10^−5^	3.8340 × 10^−5^	**COF**	0.4496	0.4847
**S/N Ratio**	−10.9842	−11.6730	**S/N Ratio**	6.9434	6.2905
**% Error**	**7.624**		**% Error**	**7.232**	

## Data Availability

The data presented in this study are available on request from the corresponding author.
